# A systematic review and meta-analysis assessing the efficacy of Tuina for nocturnal enuresis in children

**DOI:** 10.3389/fphar.2024.1421130

**Published:** 2024-06-19

**Authors:** Xin Chen, Wei-jie Sun, Jing-rong Wang, Ying-ying Cai, Xiao-dan Yu

**Affiliations:** ^1^ Department of Developmental and Behavioral Pediatrics, Fujian Children’s Hospital(Fujian Branch of Shanghai Children’s Medical Center), College of Clinical Medicine for Obstetrics & Gynecology and Pediatrics, Fujian Medical University, Fuzhou, China; ^2^ The First Clinical Medical College of Nanjing University of Chinese Medicine, Nanjing, China; ^3^ Department of Developmental and Behavioral Pediatrics, Shanghai Children’s Medical Center, School of Medicine, Shanghai Jiao Tong University, Shanghai, China

**Keywords:** Tuina, systematic review, desmopressin acetate, behavioral interventions, single-symptom nocturnal enuresis

## Abstract

**Background:** Desmopressin acetate (DDAVP) and behavioral interventions (BI) are cornerstone treatments for nocturnal enuresis (NE), a common pediatric urinary disorder. Despite the growing body of clinical studies on massage therapy for NE, comprehensive evaluations comparing the effectiveness of Tuina with DDAVP or BI are scarce. This study aims to explore the efficacy of Tuina in the management of NE.

**Methods:** A systematic search of international databases was conducted using keywords pertinent to Tuina and NE. The inclusion criteria were limited to randomized controlled trials (RCTs) that evaluated NE treatments utilizing Tuina against DDAVP or BI. This meta-analysis included nine RCTs, comprising a total of 685 children, to assess both complete and partial response rates.

**Results:** Tuina, used as a combination therapy, showed enhanced clinical efficacy and improved long-term outcomes relative to the control group. The therapeutic efficacy of Tuina was not directly associated with the number of acupoints used. Instead, employing between 11 and 20 acupoints appeared to have the most significant effect.

**Conclusion:** The findings of this meta-analysis support the potential of Tuina as an adjunct therapy to enhance the sustained clinical efficacy of traditional treatments for NE. However, Tuina cannot completely replace DDAVP or BI in the management of NE. While this study illuminates some aspects of the effective acupoint combinations, further research is crucial to fully understand how Tuina acupoints contribute to the treatment of NE in children.

**Systematic Review Registration:** https://www.crd.york.ac.uk/PROSPERO/display_record.php?RecordID=442644, identifier CRD42023442644.

## 1 Introduction

Nocturnal enuresis (NE) is characterized by recurrent involuntary urination during sleep in children aged 5 years and older, persisting for over 3 months with at least two episodes per week. This condition, resulting from the child’s inability to awaken from sleep (Austin et al., 2016), exhibits a prevalence rate ranging from 4.8% to 15.2%, which notably declines with advancing age (Ferrara et al., 2020). Moreover, NE significantly impacts the psychological wellbeing and overall quality of life of affected individuals (Kuoch et al., 2019).

The primary treatments for NE include desmopressin acetate (DDAVP) and behavioral interventions (BI) (Alqannad et al., 2021). While these modalities offer therapeutic benefits, their implementation is often protracted and fraught with challenges, including adverse drug reactions and a high rate of symptom recurrence post-treatment discontinuation. These factors complicate adherence for both patients and their families (Kuwertz-Bröking and von Gontard, 2018). Consequently, there is a pressing need for alternative therapeutic strategies to address the multifaceted challenges of managing NE in children.

Tuina, a recognized form of complementary and alternative medicine (CAM), has been integral to human health for centuries. In recent decades, the US Food and Drug Administration (FDA) has established regulatory guidelines for CAM practices (Food and Drug Administration, 2006). Tuina, characterized by its non-invasive, needle-free, cost-effective, and non-pharmacological approach, employs manual manipulation to enhance patient wellbeing (Wan et al., 2018). Gaining substantial popularity in Western nations (Su and Li, 2011), Tuina operates on the principle of activating meridian acupoints (Mehta et al., 2017), which are critical pressure points located superficially on the skin. The activation of these acupoints stimulates myelinated nerve fibers within the hypothalamus and pituitary gland, triggering the release of β-endorphins into the cerebrospinal fluid and bloodstream (Kwan et al., 2014). This biochemical process is instrumental in modulating physiological responses, such as inhibiting bladder contractions by elevating spinal and reflexive β-endorphin levels (Mehta et al., 2017). Previous meta-analyses have highlighted Tuina’s efficacy as a therapeutic option for pediatric NE; however, these studies often excluded trials that compared Tuina with established treatments like DDAVP or BI, which are considered standard care approaches (Tong et al., 2022). The long-term effectiveness of Tuina in treating NE remains to be fully assessed. Given the variety of techniques and the frequency of acupoint utilization, this meta-analysis seeks to delineate the specific roles of different Tuina applications through extensive subgroup analyses. The goal is to provide a comprehensive framework that supports informed decision-making in clinical settings.

## 2 Methods

This systematic review and meta-analysis were conducted in alignment with the Cochrane Collaboration guidelines and the PRISMA statement. Ethical approval was not required for this study. The protocol was submitted for registration with the International Prospective Register of Systematic Reviews (PROSPERO) on 23 July 2023 (ID: CRD42023442644); the registration was pending at the time of this manuscript’s submission.

### 2.1 Eligibility criteria

Only randomized controlled trials (RCTs) were considered for inclusion. Cohort studies, case series, and review articles were excluded. Abstracts that met the inclusion criteria were considered; however, if insufficient data were presented, the corresponding authors were contacted for additional information. Abstracts were excluded if no response was obtained. To mitigate selection bias and ensure comprehensive data analysis, all patients diagnosed with NE were included, irrespective of gender, age, educational background, or ethnicity. In the experimental arm, Tuina was administered either alone, in combination with DDAVP, or alongside BI. The control group received standard treatments, which did not include Tuina but may have included DDAVP or BI. The treatments administered to the control and experimental groups, apart from Tuina therapy, were identical. The primary endpoint was the total effective rate (TER), and the secondary endpoint assessed long-term effectiveness.

### 2.2 Search strategy

From inception through May 2023, comprehensive searches were conducted in several electronic databases including PubMed, Embase, CNKI, WANFANG DATA, and the Cochrane Library. The search terms were derived from the National Medical Library’s medical topic title synonym dictionary. A concurrent search was executed using the terms “Tuina,” “Nocturnal enuresis,” “Desmopressin acetate,” and “Behavioral intervention.” Additionally, reference lists from initially retrieved articles were reviewed to identify further studies eligible for inclusion. The inclusion criteria targeted all RCTs published in English and Chinese that assessed the efficacy of Tuina, DDAVP, and BI in the management of NE.

### 2.3 Selection and data extraction

Data extraction was conducted by two independent reviewers using a standardized form to capture information on baseline characteristics, eligibility criteria, intervention dosages, and details of experimental and control treatments, including study settings. Upon acquiring potentially relevant studies in full-text format, two authors independently assessed these for inclusion. Any disagreements were resolved through discussion among the authors. The data extraction process focused on participant numbers, demographic characteristics, treatment duration, post-treatment follow-up periods, and specific details of the massage treatment such as types of acupoints and evaluation methods employed. This data was subsequently verified by additional reviewers to ensure robust literature search, data extraction, and quality assessment processes.

### 2.4 Analyzing the risk of bias in studies included

The risk of bias in the included studies was evaluated using the Cochrane Collaboration’s risk of bias tool (Higgins et al., 2019). This comprehensive assessment addressed randomization type, allocation concealment, blinding of participants and personnel, blinding of outcome assessment, completeness of outcome data, and potential conflicts of interest such as industry support. The evaluations were independently conducted by three reviewers. In instances of differing opinions, a fourth reviewer was consulted to achieve consensus.

### 2.5 Assessing heterogeneity and recognizing reporting biases

Heterogeneity among the studies was quantified using the I^2^ statistic. A *p*-value of less than 0.05 was deemed statistically significant for heterogeneity analyses. I^2^ values of 0 indicated no observed heterogeneity, while values of 50% or higher suggested substantial heterogeneity. In cases where I^2^ exceeded 50%, subgroup analyses were conducted to explore potential sources of heterogeneity, such as variations in control types and outcome scoring systems, to maintain the accuracy of the data synthesis. Additionally, funnel plots were utilized to detect potential publication biases.

### 2.6 Data extraction

Data were collected using standardized forms and analyzed according to traditional Chinese medicine criteria for diagnosing and treating illnesses and syndromes (Su and Li, 2011). The effectiveness of the treatment was categorized into four levels: complete remission (CR), where no recurrence of enuresis was noted within a month; significantly effective (SE), characterized by a reduction in symptoms with recurrence not exceeding once per week within a month; partial remission (PR), defined by a decrease in symptom frequency but occurring more than once a week; and ineffective, where no change in symptom frequency was observed compared to baseline. The calculation of the percentages of individuals achieving CR, SE, or PR facilitated the determination of the TER.

### 2.7 Data synthesis

Data analysis was performed using Review Manager (RevMan) version 5.4 (Copenhagen: The Nordic Cochrane Centre, The Cochrane Collaboration, 2014) (Cochrane Training, 2020). Subgroup analyses were conducted to assess the efficacy of Tuina in treating NE, both in terms of short-term and long-term effectiveness, and in comparison with other therapies. One subgroup analysis focused on the efficacy of different treatment modalities within Tuina and non-Tuina populations. Another subgroup explored the relationship between the number of Tuina acupoints utilized and treatment outcomes. The commonly used Tuina acupoints, their classifications, and the combinations employed were also summarized.

## 3 Results

### 3.1 Study selection

**FIGURE 1:**
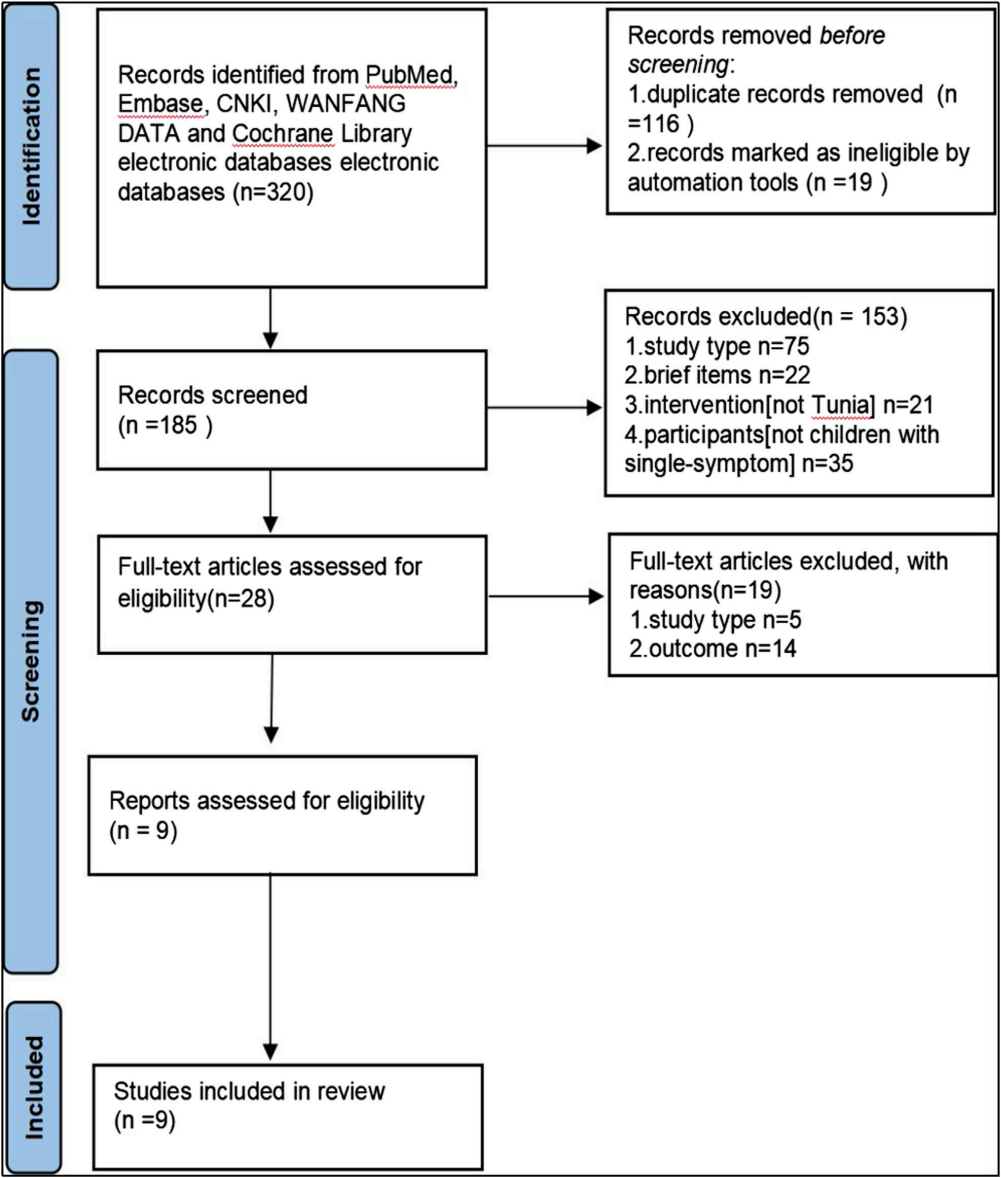
Flow chart for search results.

Eligible trials are illustrated in Figure 1, while Table 1 provides a comprehensive overview of the trials incorporated into this analysis. Nine RCTs involving 685 participants, aged between 5 and 18 years, were included. These trials featured varying sample sizes, ranging from 45 to 162 participants, and were conducted in China.

**TABLE 1:** Summarize of included studies.

Reference	Study design	Sample size (n [male/female])		Age (years, mean SD)		Intervention		Treatment duration (day)	Follow-up duration (month)	Outcomes	Acupoint
		TG	CG	TG	CG	TG	CG				
Zhang et al., 2019 (Zhang and Chen, 2019)	RCT	42,25/17	48,29/19	6.38 ± 0.59	6.18 ± 0.63	TN	DDAVP	30	6	TER,ADH, FBC,TCMJQS	*Baihui;Shenjing;Danjing;Yiniao;Laogong;Sanyinjiao;Shenshu;Dazhui;Mingmen;Dantian;Shangliao;Xialiao;Zhongliao;Ciliao;Yangchi;Quchi*
Luo et al., 2019 (Luo and Yi, 2019)	RCT	81,48/33	81,47/34	8.05 ± 1.84	8.38 ± 2.14	TD	DDAVP	90	12	TER,ADH,BVI, BC,UF,AT	*Feijing;Shending;Xiaochangjing;Yangchi;Quchi;Laogong;Sanyinjiao;Baihui;Shenque;Feishu;Pishu;Er ren shang ma;Baihui*
Feng et al., 2008 (Feng and Zhao, 2008)	RCT	40,23/17	36,20/16	5-14	5-14	TN	DDAVP	30	1	TER, TCMJQS	*Changqiang;Mingmen;Pangguangshu;Shenshu;Dazhui;Ganshu*
Ding et al., 2019 (Ding et al., 2019)	RCT	30,16/14	30,17/13	7.8 ± 1.2	8.0 ± 1.3	TBI	BI	90	2	TER TCMJQS	*Pijing;Shenjing;* *Laogong;Yangchi;Quchi;Qihai;Guanyuan;Zhongji;Feishu;Pishu;Shenshu;Pangguangshu;Zusanli;Sanyinjiao;Shangliao;Xialiao;Zhongliao;Ciliao*
Li et al., 2023 (Li, 2023)	RCT	32.15/17	30,19/11	8.7 ± 2.1	8.5 ± 1.1	TN	DDAVP	30	3	TER	*Guanyuan, Qihai, Zhongji, Mingmen, Shenshu, Baliao* *Baihui, Sishencong, bladder, Jiaji,* *largeintestine, kidney top, Guiwei, Laogong, Waiguan, Zusanli, Sanyinjiao Yanglingquan*
Wen et al., 2022 (Wen and Sun, 2022)	RCT	40.21/19	40,22/18	9.06 ± 1.94	9.12± 1.88	TD	DDAVP	90	3	TER,TCMJQS, AT	*Pijing Shenjing Qihai Guanyuan* *Pishu Shenshu Ciliao Zusanli Sanyinjiao Yaoyangguan*
Lu et al., 2023 (Lu et al., 2023)	RCT	30.18/12	30,20/10	7.26± 0.84	7.41 ± 0.79	TBI	BI	180	1	TER,TCMJQS	*Shenjing Pijing* *Qihai Wailaogong* *Yangchi Quchi* *an* *Feishu Pishu* *Shenshu Pangguangshu* *Shangliao Xialiao* *Zhongliao Ciliao* *Zusanli Sanyinjiao*
Su et al., 2013 (Su et al., 2013)	RCT	25	25	5-15	5-15	TN	DDAVP	30	3	TER,UF	*Guanyuan, Qihai, Zhongji, Mingmen, Shenshu, Baliao* *Baihui, Sishencong, bladder, Jiaji,* *largeintestine, kidney top, Guiwei, Laogong, Waiguan, Zusanli, Sanyinjiao Yanglingquan*
Zhang et al., 2017 (Zhang and ZHANG, 2017)	RCT	23,13/10	22,11/11	6.34 ± 1.56	5.87± 1.79	TN	DDAVP	90	1	TER	*Danjing Shenjing Wailaogong Dantian Shenshu Baliao Changqiang* *Dazhui Sanyinjiao*

TN: tuina; DDAVP: desmopressin acetate; TD: tuina plus desmopressin acetate; FBC: functional bladder capacity; BC: bladder capacity; TBI: tuina plus behavioral intervention; BI: behavioral intervention; UF: urinary frequency; AT: arousal threshold; TER: total effect rate; TCMGQS: Traditional Chinese medicine grading quantitative scoring. BVI: Bladder volume index. ADH: antidiuretic hormone.

### 3.2 Risk of bias assessment

**FIGURE 2:**
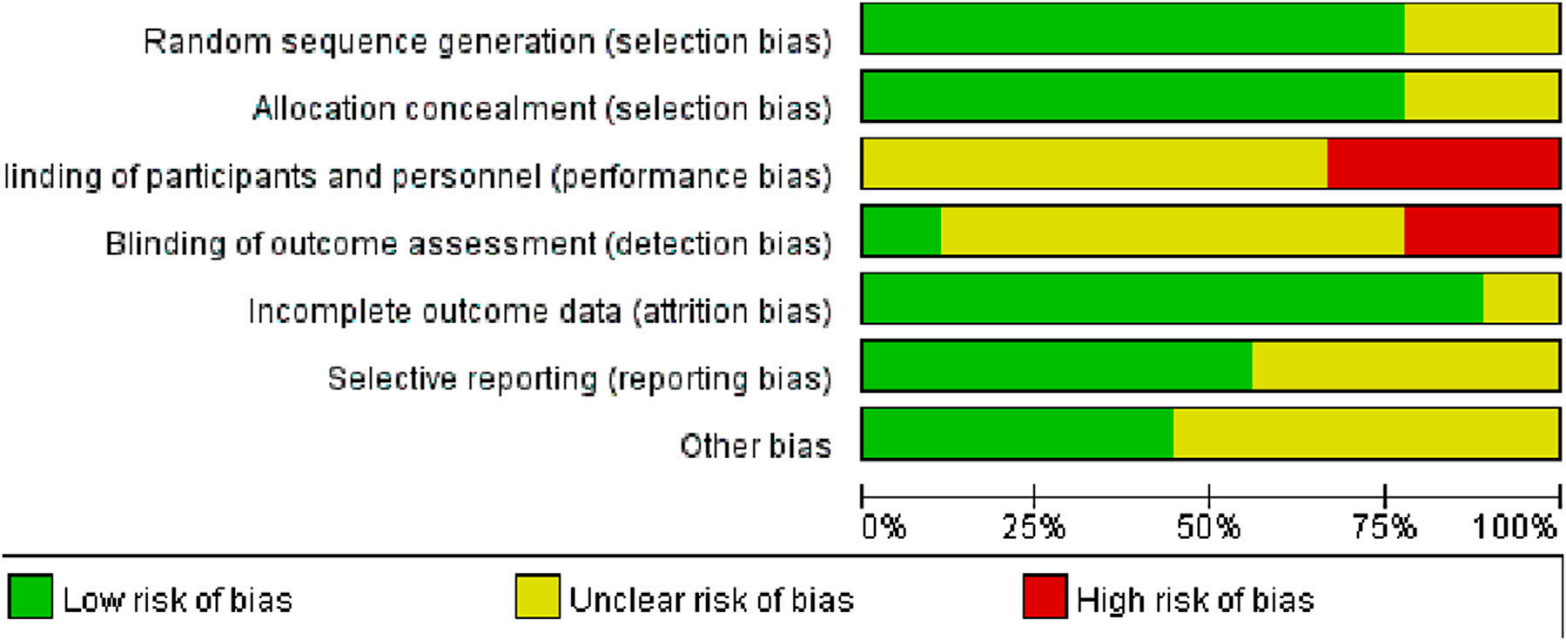
Assessment of methodological quality of randomised trials. 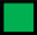 Low risk of bias; 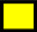 unclear risk of bias; 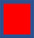 high risk of bias.

The methodologies employed across studies demonstrated considerable variability in quality (Figure 2; Table 2). The methodological shortcomings identified include unclear sequence generation in two studies, unclear risks associated with allocation concealment in two studies, unclear (six studies) or high risks (three studies) of bias in blinding of participants and personnel, and unclear (six studies) or high risks (two studies) in blinding of outcome assessments. Additionally, one study presented unclear risks regarding incomplete outcome data, and five RCTs had unclear risks related to selective reporting. Risks of other biases were unclear in five studies.

**TABLE 2:** Assessment of methodological quality of randomized trials.

	Random sequence generation (selection bias)	Allocation concealment (selection bias)	Blinding of participants and personnel (performance bias)	Blinding of outcome assessment (detection bias)	Incomplete outcome data (attrition bias)	Selective reporting (reporting bias)	Other bias
Ding 2019	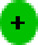	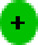	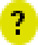	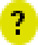	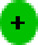	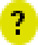	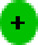
Feng 2008	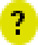	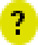	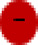	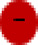	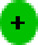	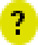	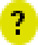
Li 2023	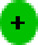	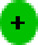	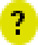	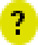	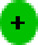	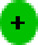	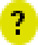
Lu 2023	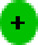	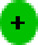	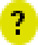	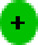	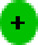	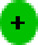	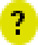
Luo 2019	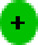	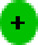	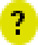	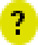	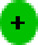	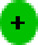	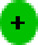
Su 2013	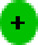	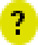	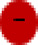	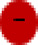	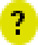	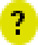	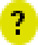
Wen 2022	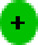	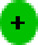	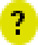	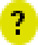	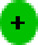	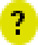	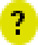
Zhang 2017	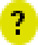	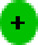	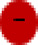	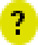	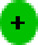	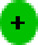	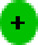
Zhang 2019	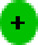	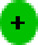	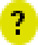	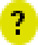	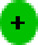	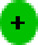	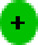

### 3.3 Total effective rate

**FIGURE 3:**
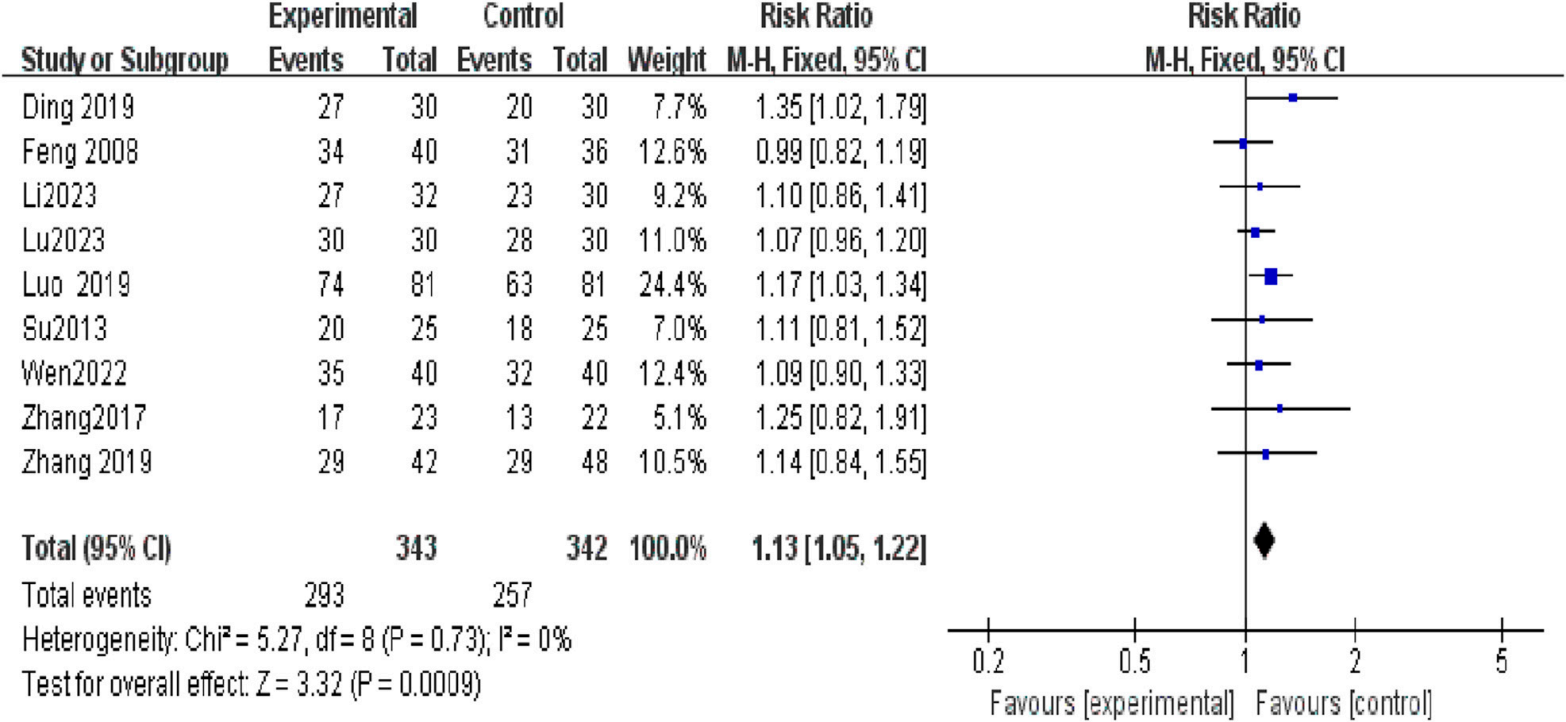
Compared to the control group, the experimental group demonstrated a substantial improvement in the total effective rate.

**FIGURE 4:**
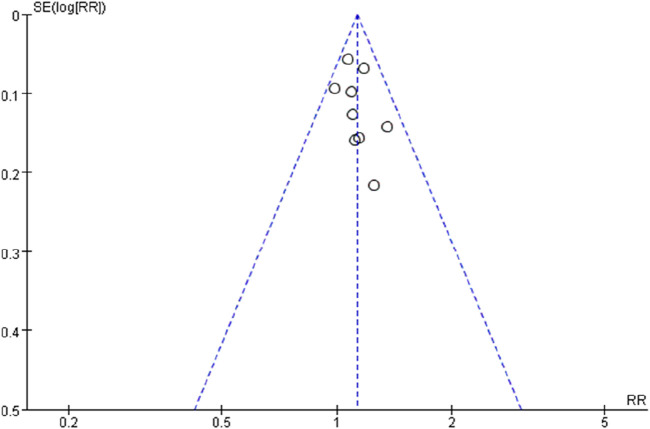
Funnel plot of comparision:risk difference of total effect rate.

The efficacy of Tuina compared with DDAVP and BI for treating NE was assessed across nine studies involving 685 participants (Feng and Zhao, 2008; Su et al., 2013; Zhang and ZHANG, 2017; Ding et al., 2019; Luo and Yi, 2019; Zhang and Chen, 2019; Wen and Sun, 2022; Li, 2023; Lu et al., 2023). No significant heterogeneity was observed among the studies (I^2^ = 0), prompting the use of a fixed-effects model for the meta-analysis. The results indicated a significantly higher TER in the Tuina treatment group compared to the control group, with a relative risk (RR) of 1.13 (95% confidence interval [CI)], 1.05 to 1.22; *p* < 0.0009) (Figure 3). Additionally, funnel plots used to assess publication bias showed no evidence of bias (Figure 4).

### 3.4 Short-term and long-term treatment durations analysis

**FIGURE 5:**
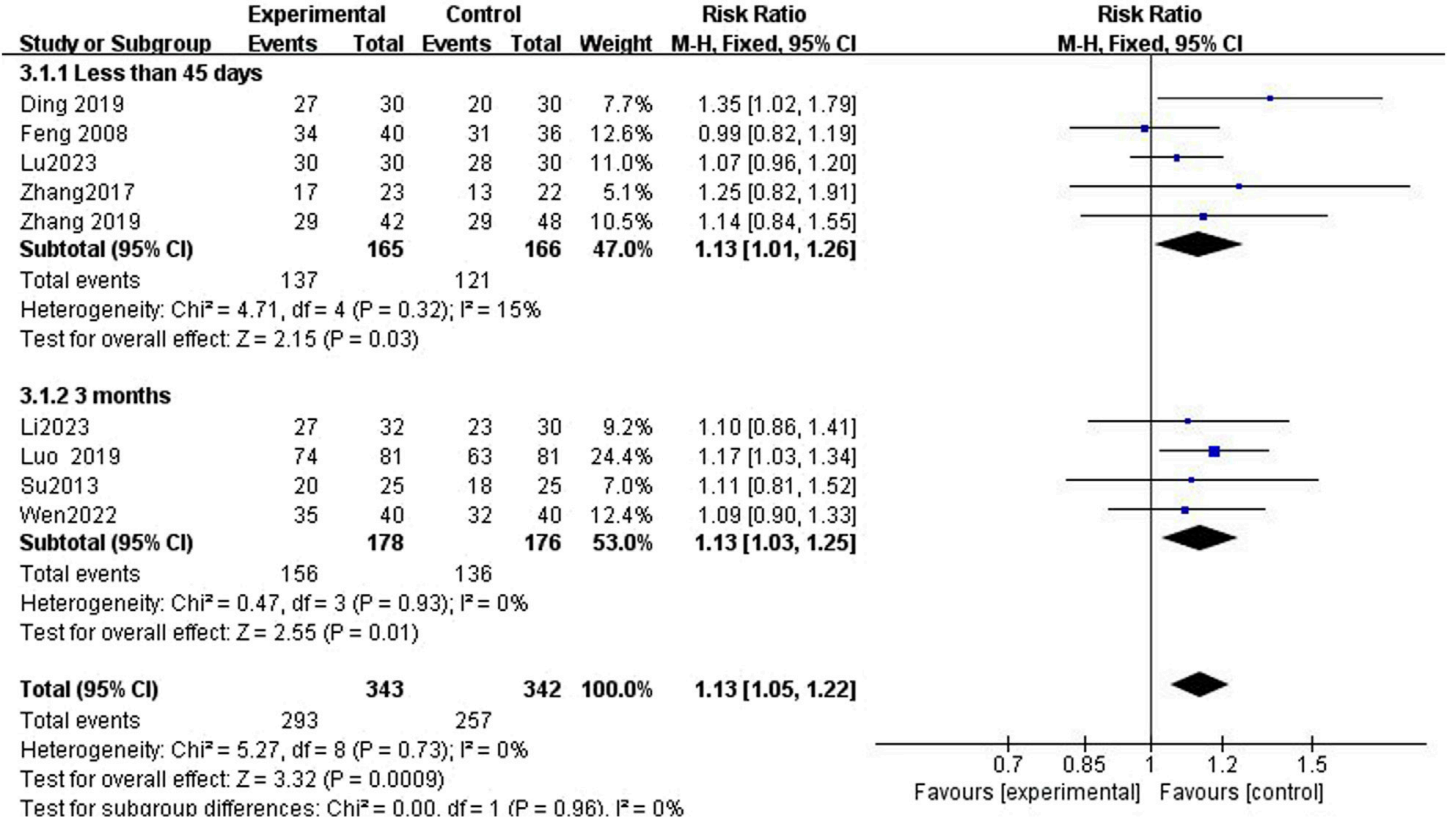
In subgroup analysis, the experimental group demonstrated a significantly improved total effective rate compared to the control group.

Subgroup analyses revealed a significantly higher TER in the Tuina treatment group compared to the non-Tuina control group. For short-term treatments (less than 45 days), the RR was 1.13 (95% CI, 1.01 to 1.26; *p* = 0.03); and for long-term treatments (3 months), the RR was 1.13 (95% CI, 1.03 to 1.25; *p* = 0.01) (Figure 5).

### 3.5 Tuina *versus* non-Tuina treatment analysis

**FIGURE 6:**
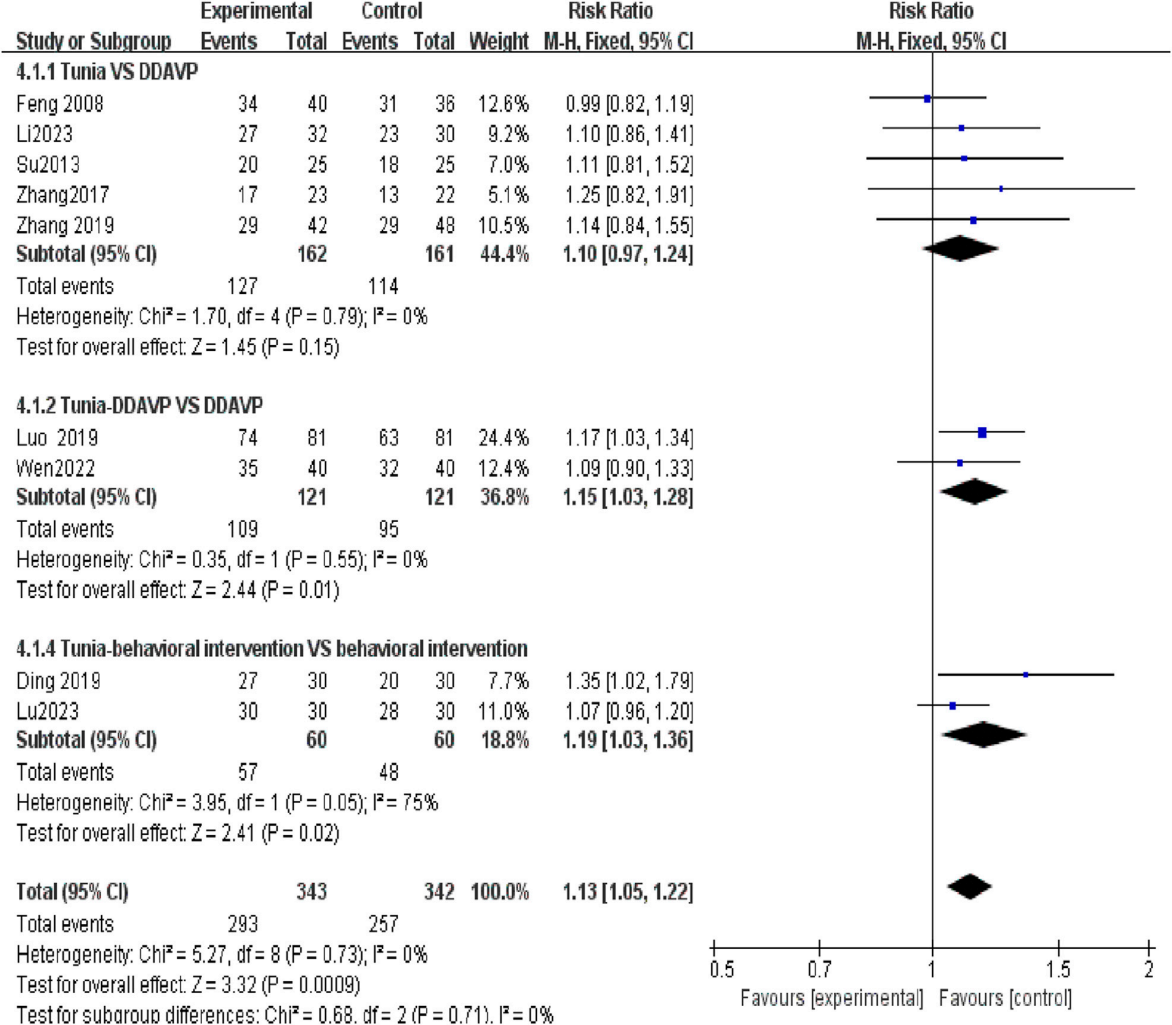
In subgroup analysis, the efficacy of Tuina as a monotherapy is inferior to that of an adjuvant treatment.

Five studies (Feng and Zhao, 2008; Su et al., 2013; Zhang and ZHANG, 2017; Zhang and Chen, 2019; Li, 2023) investigated the efficacy of Tuina for treating NE in children compared to DDAVP alone. The analysis showed no statistically significant difference in TER between Tuina alone and the DDAVP group, with an RR of 1.10 (95% CI, 0.97 to 1.24; *p* = 0.15). However, in two studies (Luo and Yi, 2019; Wen and Sun, 2022), the combination of Tuina with DDAVP exhibited a higher TER compared to DDAVP alone, reaching statistical significance (RR = 1.15; 95% CI, 1.03 to 1.28; *p* = 0.01). Additionally, two studies (Ding et al., 2019; Lu et al., 2023) compared the TER of Tuina combined with BI against BI alone, showing an RR of 1.19 (95% CI, 1.03 to 1.36; *p* = 0.05) (Figure 6).

### 3.6 Acupoint in the selected studies

**FIGURE 7:**
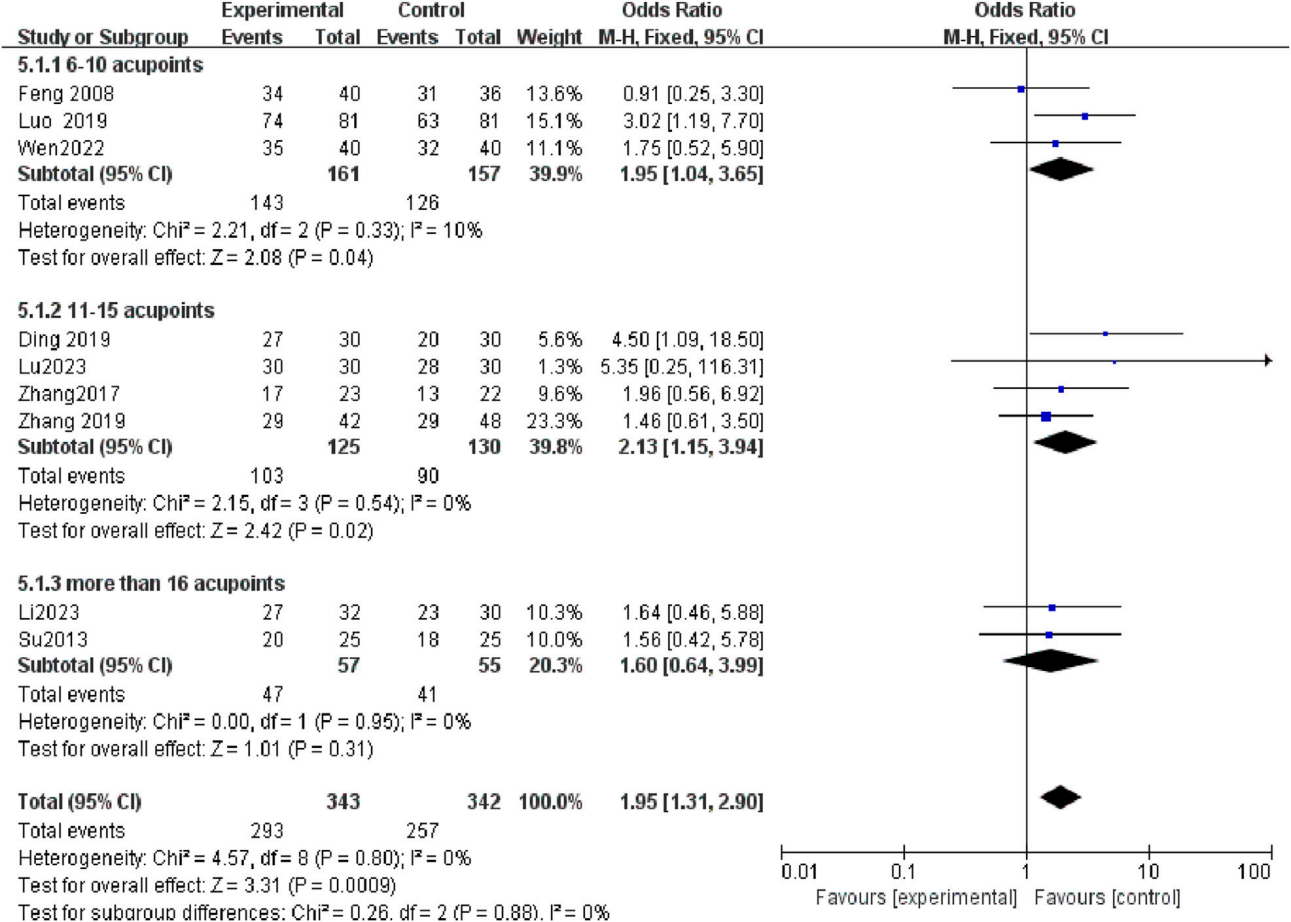
In subgroup analysis, the most notable therapeutic benefit is evident in Tuina when utilizing 11–20 acupoints.

In Tuina therapy, the conventional wisdom that “more acupuncture points yield better results” is not supported by our findings. Subgroup analyses suggest that employing 11–20 acupoints tends to produce the most pronounced effects (Figure 7). The most frequently used acupoints were San Yin Jiao (SP6) and Shen Shu (BL23) (Table 3). Other commonly applied acupoints included Bai Hui (GV20), Yang Chi (TE4), Qu Chi (LI11), Pang Guang Shu (BL28), Lao Gong (PC8), Pi Shu (BL20), Shang Liao (BL31), Xia Liao (BL34), Zhong Liao (BL33), Ci Liao (BL32), Chang Qiang (GV1), Zu San Li (ST36), Da Zhui (GV14), Guan Yuan (CV4), Fei Shu (BL13), Ming Men (GV4), Qi Hai (CV6), Gan Shu (BL18), Shen Que (CV8), Si Shen Cong (EX-HN1), Jia Ji (EX-B2), Yan Ling Quan (GB34), Yao Yang Guan (GV3), Wai Guan (TE5), Tian Shu (ST25), Wai Lao Gong (EX-UE8).

**TABLE 3:** Acupoint in included studies.

Study acupoint	2022	2023	2019	2019	2008	2019	2013	2023	2017	Proportion(%)
	Wen	Lu	Zhang	Luo	Feng	Ding	Su	Li	Zhang	
SP6	+	+	+	+		+	+	+	+	88.90
BL23	+	+	+		+	+	+	+	+	88.90
GV20			+	+			+	+		44.40
TE4		+	+	+		+			+	55.60
LI11		+	+	+		+			+	55.60
BL28		+			+	+	+	+		55.60
PC8							+	+		22.20
BL20	+	+		+		+				44.40
BL31		+	+			+	+	+	+	66.70
BL34		+	+			+	+	+	+	66.70
BL33		+	+			+	+	+	+	66.70
BL32	+	+	+			+	+	+	+	77.70
GV1			+		+				+	33.30
ST36	+	+				+	+	+		55.60
GV14			+		+				+	33.30
CV4	+	+				+	+	+		55.60
BL13		+		+		+				33.30
GV4					+		+	+		33.30
CV6	+	+				+	+	+		55.60
BL18					+					8.30
CV8				+						8.30
GV3	+									8.30
EX-HN1							+	+		16.70
EX-B2							+	+		16.70
GB34							+	+		16.70
TE5							+	+		16.70
EX-UE8		+	+	+		+			+	55.60
Shenjing	+	+	+			+			+	55.60
Shending				+			+	+		33.30
Pijing	+	+				+				33.30
Errenshangma				+						8.30
Feijing				+						8.30
Danjing			+						+	16.70
Xiaochangjing				+						8.30
Dantian			+						+	16.70
Guiwei							+	+		16.70
BL25							+	+		16.70

The World Health Organization’s alphanumeric code for acupuncture points is used in this table.

Chinese National Standards GB/T 12346-2021 (naming and positioning of acupuncture points) and GB/T 40997-2021 (naming and positioning of extraordinary acupuncture points).

**FIGURE 8:**
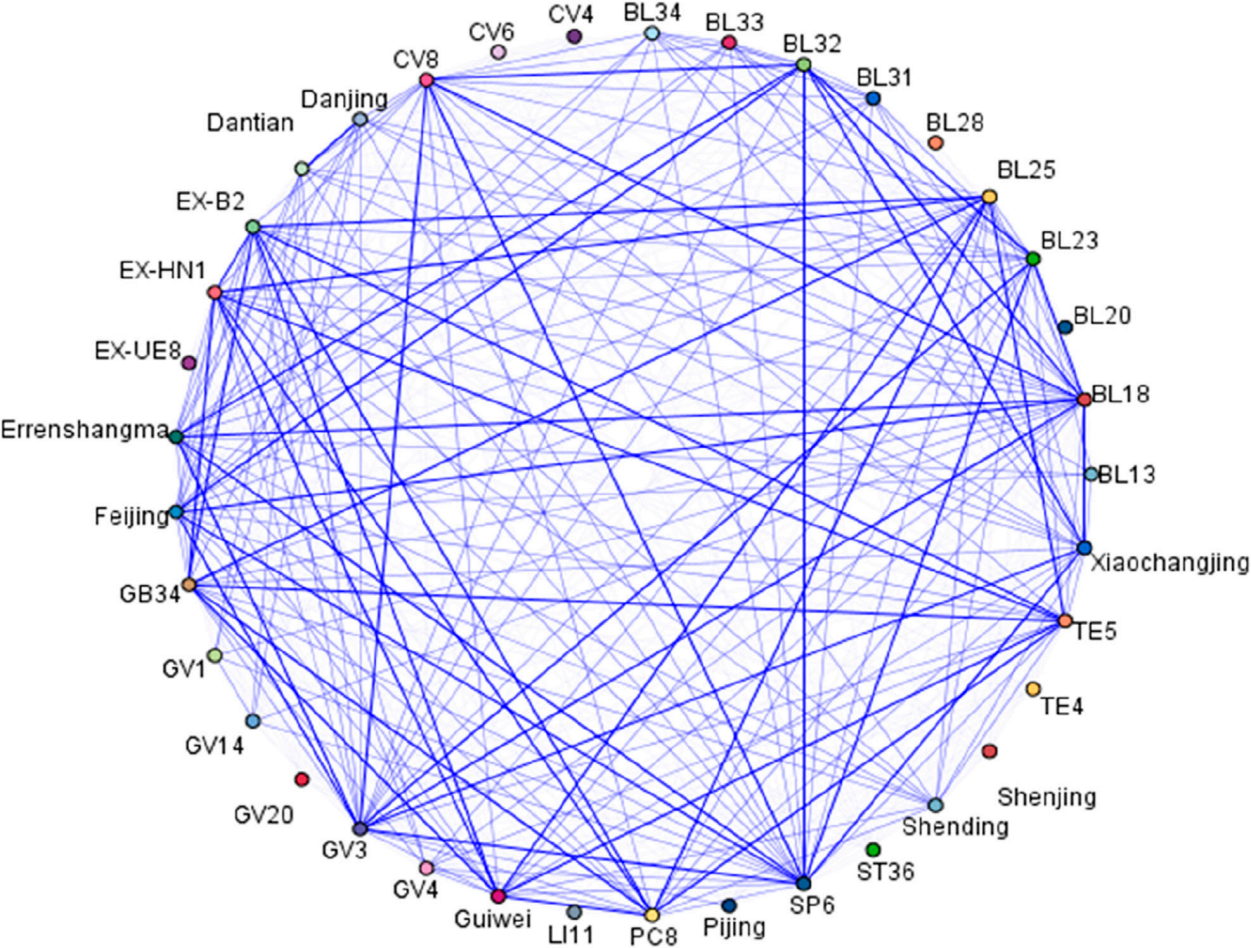
Rrepattern of high-frequency acupoints.

**FIGURE 9:**
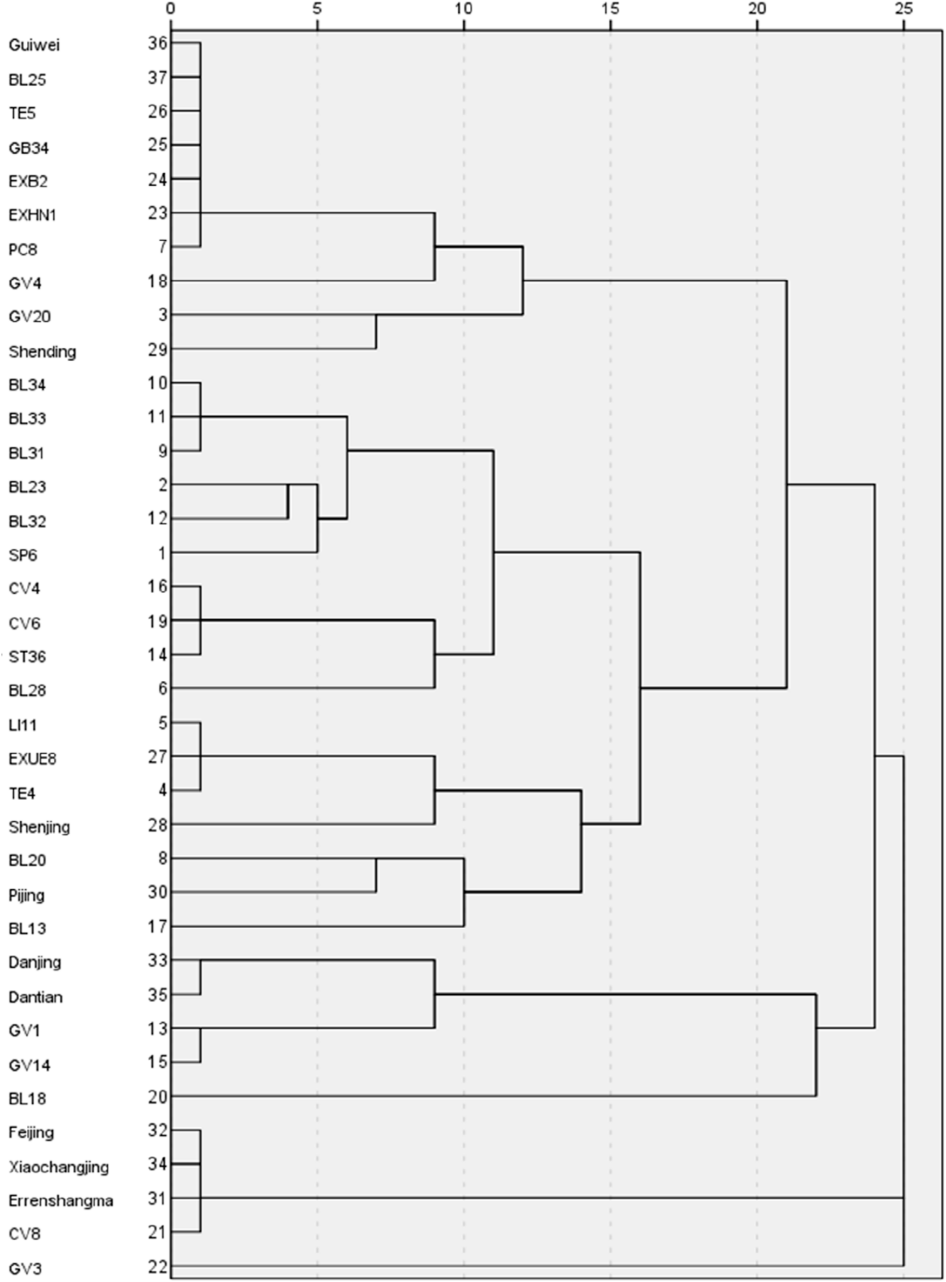
Dendrogram using Average Linkage (Between Groups) Rescaled Distance Cluster Combine.

Figure 8 illustrates the relationships between these acupoints in this study, with thicker lines indicating stronger associations. In the association analysis, the confidence percentage for the association of (Sanyinjiao, Shenshu, Baliao) BL23, SP6, and Baliao was the highest at 85.71%. The acupoints used in Tuina treatment for NE are broadly classified into six categories, with the combination of first and second groups reflecting traditional acupoint in prior research (Yuksek et al., 2003). These groupings are: 1) Guiwei/BL25/TE5/GB34/EXB2/EXHN1/PC8/GV4/GV20/Shengding; 2) BL34/BL33/BL31/BL23/BL32/SP6/CV4/CV6/ST36/BL28/LI11/EXUE8/TE4/Shenjing/BL20/Pijing/BL13; 3)Danjing/Dantian/GV1/GV14; 4)BL28; 5)Feijing/Xiaochangjing/Errenshangma/CV8; 6) GV3 (Figure 9).

### 3.7 Adverse events

Treatment-related adverse events were recorded in two of the studies reviewed (Feng and Zhao, 2008; Li, 2023), and the remaining seven studies (Su et al., 2013; Zhang and ZHANG, 2017; Ding et al., 2019; Luo and Yi, 2019; Zhang and Chen, 2019; Lu et al., 2023) observed no adverse reactions.

## 4 Discussion

This systematic review represents a first study to evaluate the efficacy of Tuina in RCTs for pediatric NE, using DDAVP and BI as control. The review encompassed data from nine RCTs involving 658 participants. It was noted that all studies in the experimental group used Tuina, either as a standalone treatment or in combination with DDAVP or BI. The findings suggest that Tuina, particularly when combined with other treatments, offers substantial benefits in enhancing clinical efficacy and improving the long-term prognosis for children experiencing NE. Given its safety, non-invasive nature, painlessness, and ease of application, Tuina emerges as an attractive treatment modality for parents.

The pathogenesis of NE is complex, influenced by multiple factors including the disruption of the circadian rhythm of hormone release and the resultant nocturnal polyuria. These factors play significant roles in the pathophysiology of NE (Yousefichaijan et al., 2016; Keten et al., 2020; Alqannad et al., 2021).

A key pathophysiological contributor to NE is the diminished bladder functional capacity coupled with increased detrusor muscle activity during the night (Kawauchi et al., 2003). Treatments with DDAVP and BI are specifically designed to address these underlying issues (Glazener and Evans, 2000; Keten et al., 2020). Concurrently, Tuina therapy stimulates myelinated nerve fibers in the hypothalamus and pituitary gland, which are essential components in the neural regulation of urinary function (Keten et al., 2020). The selection of acupoints that impact both the spinal cord urination center and the parasympathetic nerve innervation of the urinary tract is critical (Radmayr et al., 2001; Glazener et al., 2005). By increasing β-endorphin levels in the cerebrospinal fluid, Tuina may help to inhibit bladder contractions (Su and Li, 2011; Kwan et al., 2014).

Further evidence supporting the long-term efficacy of Tuina comes from four studies (Su et al., 2013; Luo and Yi, 2019; Wen and Sun, 2022; Li, 2023), which demonstrate that its therapeutic benefits persist for at least 3 months post-treatment. This data underscores Tuina’s potential to enhance the management of NE symptoms over extended periods.

In the RCTs investigating the efficacy of treating NE with Tuina and DDAVP as standalone treatments, no statistically significant outcomes were observed (Feng and Zhao, 2008; Su et al., 2013; Zhang and ZHANG, 2017; Zhang and Chen, 2019; Li, 2023). This underscores the necessity for caution in recommending Tuina as an adjunct therapy in combination with other established treatments. Tuina, with its origins in ancient Chinese medicine, is designed to correct bodily imbalances by stimulating specific acupoints along the body’s meridian system. According to traditional Chinese medicine theory, the body is viewed as a holistic network of channels and organs, and it is believed that activating these acupoints can restore balance and harmony within the organ system (Radmayr et al., 2001). Acupoints are used to influence health conditions, including NE, potentially affecting the spinal urination center and the parasympathetic innervation of the urinary tract (Chapple, 2013). The most frequently used Tuina acupoints in the treatment of NE are San Yin Jiao (SP6) and Shen Shu (BL23), with our evidence suggesting that the use of 11-20acupoints provides the most significant therapeutic effect. These acupoints are categorized into six distinct groups. Notably, two studies included in this analysis (Luo and Yi, 2019; Zhang and Chen, 2019) demonstrate an improvement in bladder function following treatment.

The study has several limitations that should be considered when interpreting the findings. First, the inclusion of studies was predominantly restricted to Chinese research, resulting in a lack of diversity in regional characteristics. Second, due to traditional practices, achieving complete double-blindness in RCTs through randomized allocation was not possible. Third, the primary outcome of overall effectiveness limited the scope of the meta-analysis. Although some studies reported on various outcome measures, including antidiuretic hormone, bladder volume index, functional bladder capacity, bladder capacity, urinary frequency, and arousal threshold (Luo and Yi, 2019; Zhang and Chen, 2019), there is a recommendation for future research to standardize data units to enhance the accuracy of Tuina’s performance evaluation in NE.

Despite facing numerous challenges, Tuina has emerged as a burgeoning CAM gaining global recognition. This study marks the first to present substantial evidence regarding the adjunctive impact of Tuina in treating NE, detailing its therapeutic methodologies and acupoint categorization. It highlights a potentially valuable complementary approach for managing NE.

## 5 Conclusion

Within the scope of adjuvant therapies for pediatric NE, Tuina demonstrates promising long-term efficacy. The acupoints most commonly employed include San Yin Jiao (SP6) and Shen Shu (BL23), with the application of 11–20 acupoints typically yielding the most significant effects. To further enhance the treatment of NE in children, extensive research is required to substantiate and standardize these methods. Although only a few reports of adverse reactions have been documented, the inherent limitations concerning study inclusion underscore the need for more comprehensive, high-quality, long-term follow-up RCTs to validate these findings.

## Figures and Tables

**Table:** 

**ADH**	Antidiuretic hormone
**AT**	Arousal Threshold
**BC**	Bladder capacity
**BI**	Behavioral intervention
**BL13**	*Feishu*
**BL18**	*Ganshu*
**BL20**	*Pi Shu*
**BL23**	*Shen Shu*
**BL28**	*Pang Guang Shu*
**BL30**	*Shang Liao*
**BL32**	*Ci Liao*
**BL33**	*Zhong Liao*
**BL34**	*Xia Liao*
**BVI**	Bladder volume index
**CAM**	Complementary and alternative medicine
**CR**	Complete remission
**CV4**	*Guanyuan*
**CV6**	*Qihai*
**CV8**	*Shenque*
**DDAVP**	Desmopressin acetate
**EX-HN1**	*Sishencong*
**EX-B2**	*Jiaji*
**EX-UE8**	*Wailaogon*
**FBC**	Functional Bladder capacity
**FDA**	Food and Drug Administration
**GB34**	*Yanlingquan*
**GV1**	*Chang Qiang*
**GV3**	*Yaoyangguan*
**GV4**	*Mingmen*
**GV14**	*Da zhui*
**GV20**	*Bai Hui*
**LI11**	*Qu Chi*
**NE**	Nocturnal enuresis
**PC8**	*Lao Gong*
**PR**	partial response
**RCTs**	Randomized controlled trials
**SP6**	*San Yin Jiao*
**SR**	Significantly effective rate
**ST25**	*Tianshu*
**ST36**	*Zu San Li*
**TBI**	Tuina plus Behavioral Intervention
**TCMGQS**	Traditional Chinese medicine grading quantitative scoring
**TD**	Tuina plus Desmopressin Acetate; TE4 *Yang Chi*
**TE5**	*Waiguan*
**TER**	total effective rate
**TN**	Tuina
**UF**	Urinary frequency

## Data Availability

The original contributions presented in the study are included in the article/supplementary material, further inquiries can be directed to the corresponding author.
